# Cancer immunotherapy comes of age and looks for maturity

**DOI:** 10.1038/s41467-020-17140-5

**Published:** 2020-07-03

**Authors:** Amanda Finck, Saar I. Gill, Carl H. June

**Affiliations:** 1 Center for Cellular Immunotherapies, Perelman School of Medicine, University of Pennsylvania, Philadelphia, PA USA; 2 Parker Institute for Cancer Immunotherapy, University of Pennsylvania, Philadelphia, PA USA

**Keywords:** Cancer therapy, Cancer immunotherapy

## Abstract

As Nature Communications celebrates a 10-year anniversary, the field has witnessed the transition of cancer immunotherapy from a pipe dream to an established powerful cancer treatment modality. Here we discuss the opportunities and challenges for the future.

## Improving the natural immune system through immune checkpoint blockade interference

**Fig. 1:**
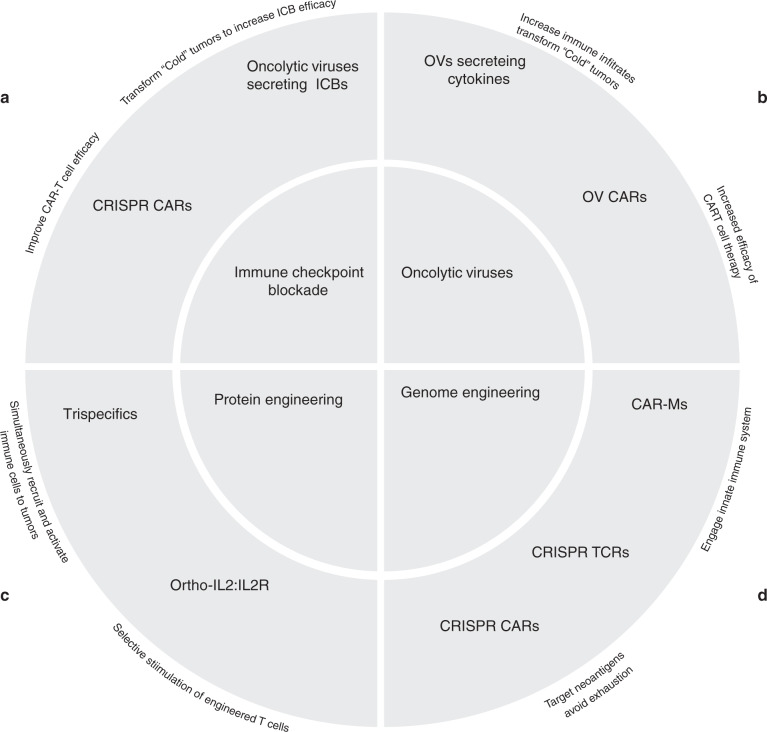
Overview of new tools immunotherapeutic developments and multiplexed approaches to improve their efficacy. **a** Immune checkpoint blockade can be augmented with oncolytic viruses and CAR T cells. **b** Oncolytic viruses can be engineered to secrete cytokines or combined with engineered T cells. **c** Advancements in protein engineering have allowed the development of trispecifics, orthogonal cytokine receptor pairs, which allow for selective stimulation of these engineered T cells. **d** Genome engineering has produced CAR-Ms and genome editing with CRISPR allow for multiple editing of multiple genes in T cells.

Advances in the understanding of basic immunology have ushered in two major approaches for cancer therapy over the past 10 years. The first is checkpoint therapy to augment the function of the natural immune system. The second uses the emerging discipline of synthetic biology and the tools of molecular biology and genome engineering to create new forms of biological structures, engineered viruses and cells with enhanced functionalities (Fig. 1).

Trastuzumab, the monoclonal antibody targeting HER2, was the first recombinant antibody to be commercially approved, in 1997. However, over the past 10 years, a paradigm shift in cancer therapy occurred with the addition of inhibitors of the immune checkpoint blockade (ICB), CTLA-4 and PD-1, to the clinics. The first to be approved was the anti-CTLA-4 inhibitor ipilimumab for metastatic melanoma in 2011. More than a dozen types of cancer are now treated with ICBs. Perhaps most surprising to the field is that lung cancer can now be treated with PD-1 (CD279) and CTLA-4 (CD152) antagonists as a component of first line therapy. 10 years ago, the consensus among thoracic oncologists was that the solution for lung cancer was inhibitors of oncogene-driven signaling pathways and the field had dismissed the notion that lung cancer would be an immunologically responsive tumor. However, between the concepts of neo-antigens and the predictive relationship of tumor mutational burden and ICB responsiveness, the response of lung cancer to immunotherapy seems less surprising in retrospect.

In the coming decade, the main challenge with ICBs is to improve efficacy, seeing that the majority of patients are resistant to therapy. Analysis of solid tumors and the tumor microenvironment (TME) have shown that responses are limited to patients with “hot” tumors, with prominent CD8+ T cell infiltrates. Contrarily, TGFβ has emerged as a dominant mechanism of resistance to ICB in “cold” tumors, tumors that are devoid of immune infiltrates^1^. The major challenge will be developing strategies for ICB to address all types of cancer histologies. While work is underway in attempt to better understand the underlying mechanisms and to convert cold to inflammatory tumors, other issues still remain. Even in demonstrated cases of efficacy, ICBs have also been associated with autoimmune toxicities, further warranting the development of approaches to thwart these adverse events. Thus, the major goal of future immunotherapeutic efforts will be geared towards eliminating this glass ceiling of efficacy currently observed for ICBs.

## Advances in synthetic biology

The past decade has witnessed the first commercial approvals of engineered viruses and cells for cancer therapy. Ongoing investment in the biotechnology industry, manifesting in a robust clinical trial portfolio, raises optimism that many new forms of cell and gene therapy will be approved in the coming decade. The US Food and Drug Administration (FDA) has predicted that new cell and gene therapy products will be approved at a rate of 10–20 new products per year by 2025.

## Engineered virotherapy

Based on “experiments of nature”, where tumor regressions were observed in patients with viral infections, viruses have been engineered for decades to be selectively oncolytic for cancer cells or as a means of immunotherapy^2^. The first and only oncolytic virus (OV) to reach FDA approval for cancer therapy was talimogene laherparepvec, an engineered herpes virus in 2015. Of more recent interest are OVs which selectively invade and replicate within tumor cells to induce immunogenic cell death. In addition to serving as a method to selectively kill tumor cells, OVs have been employed to transform “cold” tumors to increase the efficacy of PD-1/PD-L1 blockades. Wang et al. and colleagues^3^, were able to induce CD8+ T cell dependent systemic antitumor activity against dominant and subdominant neoantigen epitopes in mouse models of cancer by engineering OVs to secrete selective PD-L1 inhibitors and GM-CSF. Their work addressed the challenge of low mutational burdens and neoantigen loads, as well as poor MHC presentation and poor T cell infiltration seen in “cold” tumors and has potential as both a monotherapeutic or in combination with other immunotherapies. There are many strategies to test OVs that transform “cold” tumors to inflamed tumors^4^. Biosafety considerations have been a practical challenge to the development of OVs due to their potential to spread and the potential for recombination with wild-type viruses. The major scientific challenge to the field is to overcome the immunogenicity of OVs so that they can replicate more extensively in vivo and be readministered as needed.

## Protein engineering

During the past 10 years, antibodies and other proteins made by recombinant DNA technology came to dominate the pharmaceutical industry as 7 of the top 10 selling drugs in the US were engineered proteins in 2018. There are hundreds of monoclonal antibodies currently in clinical trials, however during the next decade, advances in protein engineering will likely lead the field beyond monoclonal antibodies. First, various bispecific antibodies have demonstrated improved activity over standard monoclonal antibodies. For example, the FDA granted approval to blinatumomab, a bispecific anti-CD3 and anti-CD19 antibody for use in the treatment of relapsed or refractory B-cell precursor ALL in 2014. A major issue currently debated is the choice of bispecific reagents versus chimeric antigen receptor T (CAR-T) cells. Bispecific antibodies have the advantage of a lower cost of manufacturing. However, it remains to be seen whether they can serve as a stand-alone therapy for advanced leukemia or serve as a bridge to a costlier procedure such as a hematopoietic transplant. Most recently, trispecific antibody technologies have been developed. It is likely that the molecule in the first clinical trial to test this technology will have specificities for CD38, CD3 and CD28, with the goal of simultaneously enhanced T cell activation through CD3 and CD28 and direct targeting of myeloma cells through CD28 and CD38^5^.

Another major advancement in the area of protein engineering was methods developed to achieve receptor-ligand orthogonalization. IL-2 is the principal growth factor for T cells and was approved by the FDA in 1992 for metastatic kidney cancer. However, a major limitation to the use of IL-2 has been deleterious effects associated with the IL-2 dependent expansion of regulatory T cells that diminishes the therapeutic effect. Sockolosky et al. engineered T cells to express an orthogonal mutant IL-2 receptor (ortho-IL2R) such that an orthogonal mutant IL-2 (ortho-IL2) would only activate the ortho-IL2R expressed by the engineered T cells^6^. In preclinical studies, this system resulted in increased tumor killing, less dysfunctional T cells, and did not increase regulatory T cells. These promising results suggest orthogonal pairs of engineered cytokines and cytokine receptors can specifically activate engineered T cells, such as CAR T cells or T cell receptor (TCR)-T cells, while ignoring bystander T cells, thereby enhancing their overall durability and efficacy. This may ultimately overcome the challenge of systemic IL-2 administration and may enable the use of other cytokines that have been virtually abandoned for reasons of toxicity, such as IL-12 and IL-10.

## Chimeric antigen receptor T cells

Ex vivo modification of immune effector cells (IECs) can endow them with novel specificity and functions. T cells, and more recently natural killer cells, can be removed from the blood of patients or healthy donors and genetically engineered to express CARs that target cell-surface molecules on cancer cells. The first CAR T cells to be tested in humans with hematologic malignancies targeted the B cell antigen CD20, but it was not until a second generation of CARs, incorporating built-in costimulation via CD28 or CD137 and against CD19, that CAR T cells showed their true potential^7^. Licensing by the FDA of tisagenlecleucel for pediatric B-ALL in 2017, and of axicabtagene ciloleucel and tisagenlecleucel for non-Hodgkin lymphoma in 2018 represented a landmark in synthetic biology and spurred an enormous wave of innovation in the spheres of research, clinical practice, manufacturing, and translational immunology.

Engineered cells are a living drug with non-traditional mechanisms of action, pharmacokinetics, toxicities and efficacy. A single administration can lead to a profound anti-tumor effect with cases of years-long persistence, allowing for potential immunosurveillance. In one remarkable case leukemia eradication was shown to occur from the progeny of a single T cell^8^. Conversely, the strong immune selection pressure can lead to antigen-negative relapse of leukemia, illuminating the mechanism of action of CAR T cells and heralding the need for multi-specific CARs or other combinations^9^. The emergence of novel toxicities such as cytokine release syndrome (CRS) and immune effector cell-associated neurotoxicity syndrome (ICANS) has also necessitated the development of mitigation and treatment strategies, and to date the administration of IECs remains the province of specialized institutions. CD19 and other B-cell lineage antigens, including BCMA, are equally present on normal and malignant B cells. The challenge to the field over the next decade will be to engineer CAR T cells that are effective for solid tumors.

## Genome engineering

Advancements in CRISPR-Cas9 have presented opportunities to overcome the obstacles faced by CAR T cell therapies in both hematologic and solid tumors. While CAR technology is restricted to cell membrane targets (largely lineage antigens), replacement of the endogenous T cell receptor alpha/beta chains with a transgenic TCR can impart novel specificity towards intracellular antigens, presented on the tumor cells’ human leukocyte antigen (HLA) molecules. This is of particular interest given that most tumor-associated or tumor-specific targets are located intracellularly, but poses specific issues relating to the HLA-restricted nature of TCR-based recognition. Innovations combining cell therapy and gene editing have been applied to target acute myeloid leukemia or T cell lymphomas and leukemias^10^.

In the solid TME such advancements in CRISPR-Cas9 can be leveraged to target exhaustion, anergy, and potentially senescence. Multiple groups have demonstrated the utility of CRISPR-Cas9 in targeting multiple genes. Most recently, Stadtmauer et al. used CRISPR-Cas9 to knock out endogenous *TRAC, TRBC*, and *PDCD1* while transducing a NY-ESO-1 TCR in order to increase the expression of the NY-ESO-1 directed TCR and potential avoid T cell exhaustion^11^. The induction of T cell exhaustion, particularly in solid tumor immunotherapy is currently thought to be the central challenge to improving response rates^12^. The combination of multiplex human genome engineering with cell therapy has the potential to reverse or prevent the induction of T cell exhaustion. Senescence, a related but distinct entity from exhaustion, is increasingly thought to have a role in the resistance to tumor immunotherapy but has been difficult to study since it is not well modeled in mice^13^.

Continuing with the trend of synthetic biology, the engineering of other immune cells, such as stem cells, NK cells and macrophages, is likely to gain increasing attention. CAR NK cells appear safe and have antitumor activity in early stage trials^14^. Engineered CAR macrophages (CAR-Ms) were able to redirect macrophage phagocytosis in the TME and convert endogenous M2 macrophages to a pro-inflammatory M1 phenotype. The CAR-Ms ultimately demonstrated antigen-specific tumor clearance in vitro^15^. In addition to coordinating an adaptive immune response with the engineering of an innate immune cell, this work also highlights the potential to translate immune cell engineering to other hematopoietic lineages.

As cancer immunotherapeutic research continues, we expect an increasing reliance on synthetic biology. As highlighted above, these modalities also have the potential to be multiplexed with one another as a means to overcome current obstacles faced by each of them individually. Current challenges to the field also necessitate further work to better understand and circumvent tumor subclonal diversity, T cell exhaustion and senescence in the context of the hostile TME. It is likely that the field will achieve substantial progress during the next decade in the treatment of patients. The “stretch goal”, and the promise of immunotherapy that would make all this investment worthwhile, would be the ability to deliver a “one and done” treatment for cancer.
